# Supplementary material for: Clade diversification dynamics and the biotic and abiotic controls of speciation and extinction rates
Source: Nat Commun. 2018 Aug 1;9:3013. doi: 10.1038/s41467-018-05419-7 (PMC6070539; doi:10.1038/s41467-018-05419-7)
Supplement: Supplementary file 1 — Supplementary Information [file 41467_2018_5419_MOESM1_ESM.pdf]

Supplementary Information for

# Clade diversification dynamics and the biotic and abiotic controls of speciation and extinction rates

Robin Aguilée, Fanny Gascuel, Amaury Lambert, Regis Ferriere

*Nature Communications*

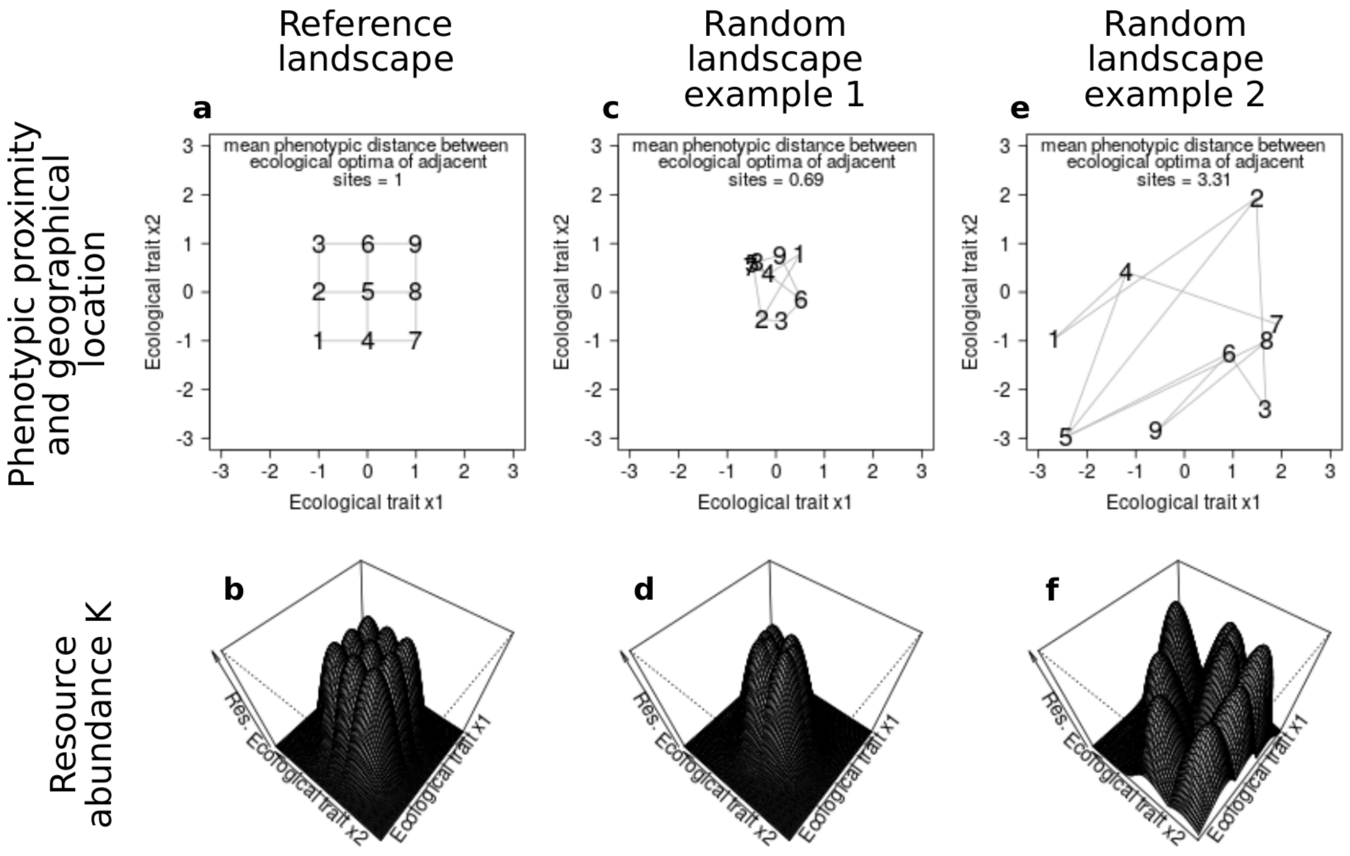


**Supplementary Figure 1: Resource distribution among geographic sites: gradient-like and random patterns.** The landscape is fragmented into nine geographic sites (numbered 1 to 9) and each site is characterized by an environmental optimum in the ecological trait space $(x_1, x_2)$. **a**-**b**, Baseline scenario in which environmental optima follow a two-dimensional gradient. The phenotypic distance between ecological optima of adjacent sites is constant (equal to 1 arbitrary unit) across the landscape. **c**-**f**, two random landscapes with different mean phenotypic distance between ecological optima of adjacent sites (**c**-**d**: 0.69 unit; **e**-**f**: 3.31 units). *Top row* (**a**, **c**, **e**): Relation between phenotypic distance between sites and their geographical distance (edges connect geographically adjacent sites). *Bottom row* (**b**, **d**, **f**): Global distribution of resources if all sites were merged together.

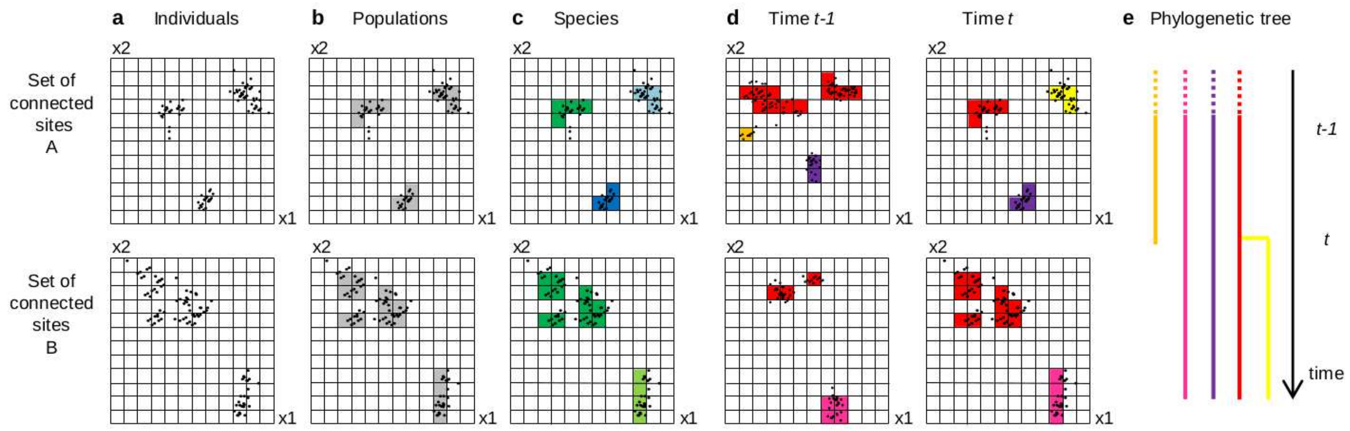


**Supplementary Figure 2: How to delineate species and follow their evolutionary history.** In this example, we consider two sets of connected geographical sites (A and B), and represent individuals (black dots) in the phenotypic space $(x_1, x_2)$, divided into cells of width $s_{x_1} \times s_{x_2}$ (**a**). We determine local populations by grouping, for each set of connected sites, individuals that occur in adjacent high density cells (defined as containing more than half the average number of individuals within non empty phenotypic cells; in grey, **b**). Then, we consider populations of all sets of connected sites (A, B,...) and group into species (same color, **c**) those that (*i*) can interbreed (mating probability above *AMT* = 0.01) and (*ii*) are not genetically incompatible (number of loci harboring incompatible alleles below *GIT*). Here, the green populations in the sets of connected sites A and B can interbreed and are not genetically incompatible; the orange and pink populations might interbreed based on their phenotypic and assortative mating traits but they are genetically incompatible; and the yellow population, even though it is not genetically incompatible with the green populations (due to recent divergence), may not interbreed with them due to phenotypic traits that are too different.

To determine the ancestry of the species $\left[S_t^{(i)}\right]_{i \in \{1,\dots N_t\}}$ delineated at time step *t*, and thus the changes in species diversity between time step *t* – *1* and time step *t*, we compare the number of loci carrying genetic incompatibilities and the phenotypic traits of species and populations at time *t* to those at time *t* – *1* (**d**). Descent relationships are determined by the minimum number of loci causing genetic incompatibilities between entities at time *t* and at time *t* – *1* and, if the latter are equal for multiple species at time *t* – *1*, by minimum Euclidian distances between their average phenotypic traits. Speciation occurs if different species $S_t^{(i)}$ descend from the same species $S_{t-1}^{(j)}$ ; in this case (*e.g.* the red and yellow species at time *t*), the species $S_t^{(i)}$ most similar (*i.e.* harboring the minimum number of genetic incompatibilities or, in case of equality, minimum phenotypic difference) to $S_{t-1}^{(j)}$ is $S_{t-1}^{(j)}$ (here the red species), whereas other species $S_t^{(i)}$ (here the yellow species) are new ones, descending from $S_{t-1}^{(j)}$. Hybridization occurs if a species $S_t^{(i)}$ includes populations descending from populations of several different species $S_{t-1}^{(j)}$ ; in that case $S_t^{(i)}$ (*e.g.* the red species at time *t*), has evolved from the most similar of its parental species $S_{t-1}^{(j)}$ (here the red species), partly due to hybridization, whereas other species $S_{t-1}^{(j)}$ (here the orange species) might go extinct. This process allows us to record all speciations, extinctions and overall changes in species diversity and composition at each time step *t* (every 100 generations; using a time step of 10 generations leads to similar relationships between species[1]) (**e**).

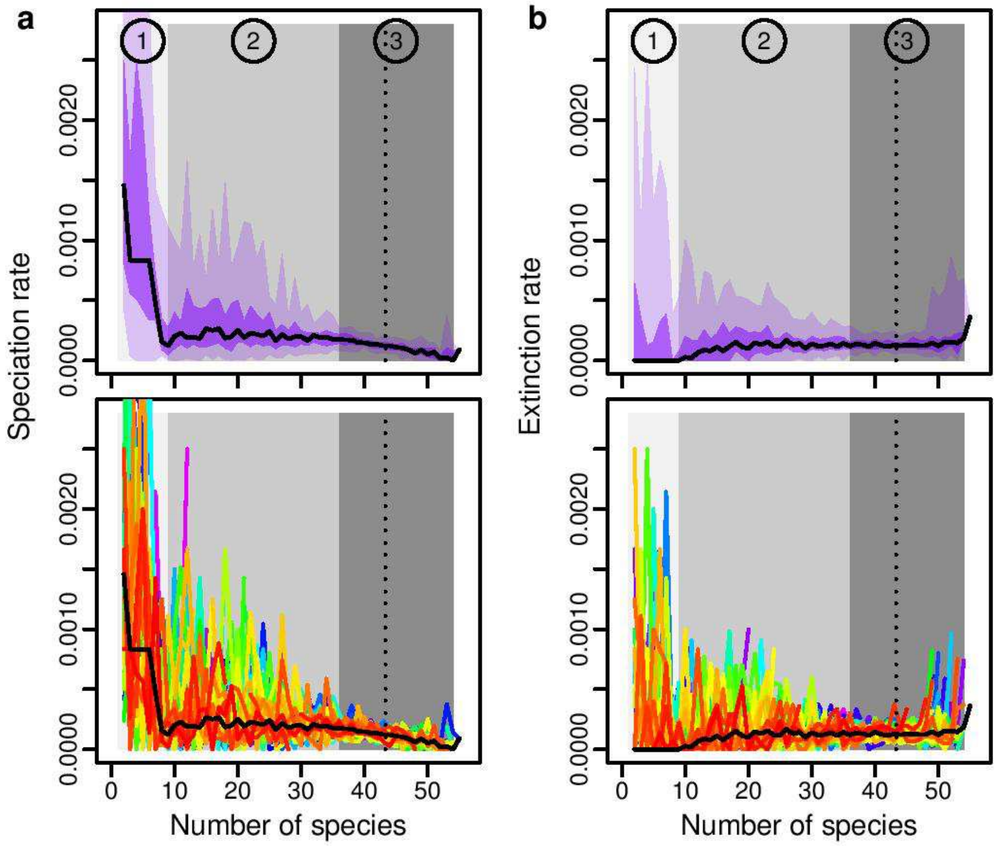


**Supplementary Figure 3: Diversity-dependence of macroevolutionary rates. a,** Diversity-dependence of speciation rates. **b**, Diversity-dependence of extinction rates. The three stages (1)-(3) of diversification are highlighted by grey shadings as in Fig. 1, and black lines give median values over 50 simulations replicates. Top row: dark and light purple areas give 95% and 50% confidence intervals, respectively. Bottom row: colored lines show data from individual simulations. Vertical dotted lines indicate the stationary species diversity. Parameter values as in Supplementary Table 1.

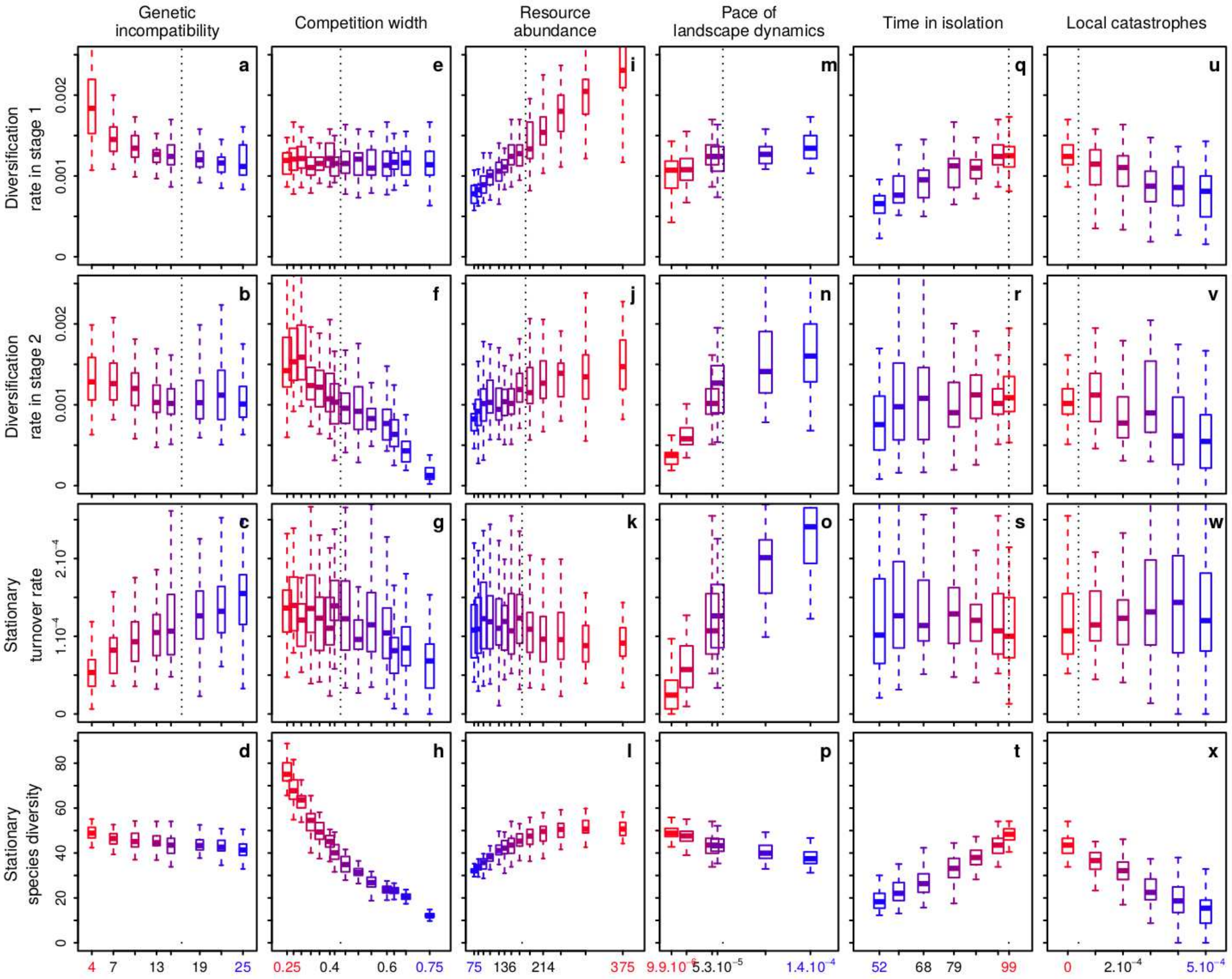


**Supplementary Figure 4: Effect of different biotic and abiotic factors on diversification rates (during stage 1 in a, and stage 2 in b), turnover rates (c) and species diversity (d) at stationary state.** Parameter values varied for each factor (from left to right): *GIT* (number of genetic incompatibility loci causing reproductive isolation; **a-d**), $\sigma_C/\sigma_K$ (scaled competition width; **e-h**), $K^*$ (carrying capacity at the environmental optimum; **i-l**), $1/(1/f + 1/c)$ (with $f$ the rate of geographic barrier arising and $c$ the rate of barrier removal; **m-p**), $f/(c + f)$ (**q-t**), and *cr* (rate of local catastrophic extinction; **u-x**). Results from different parameter values are shown on a blue to red gradient, with blue (red) corresponding to lowest (highest) species diversity at stationary state. Boxplots, calculated over 50 simulation replicates, represent the first, second and third quartiles, with whiskers giving the maximum and minimum values. Other parameter values as in Supplementary Table 1.

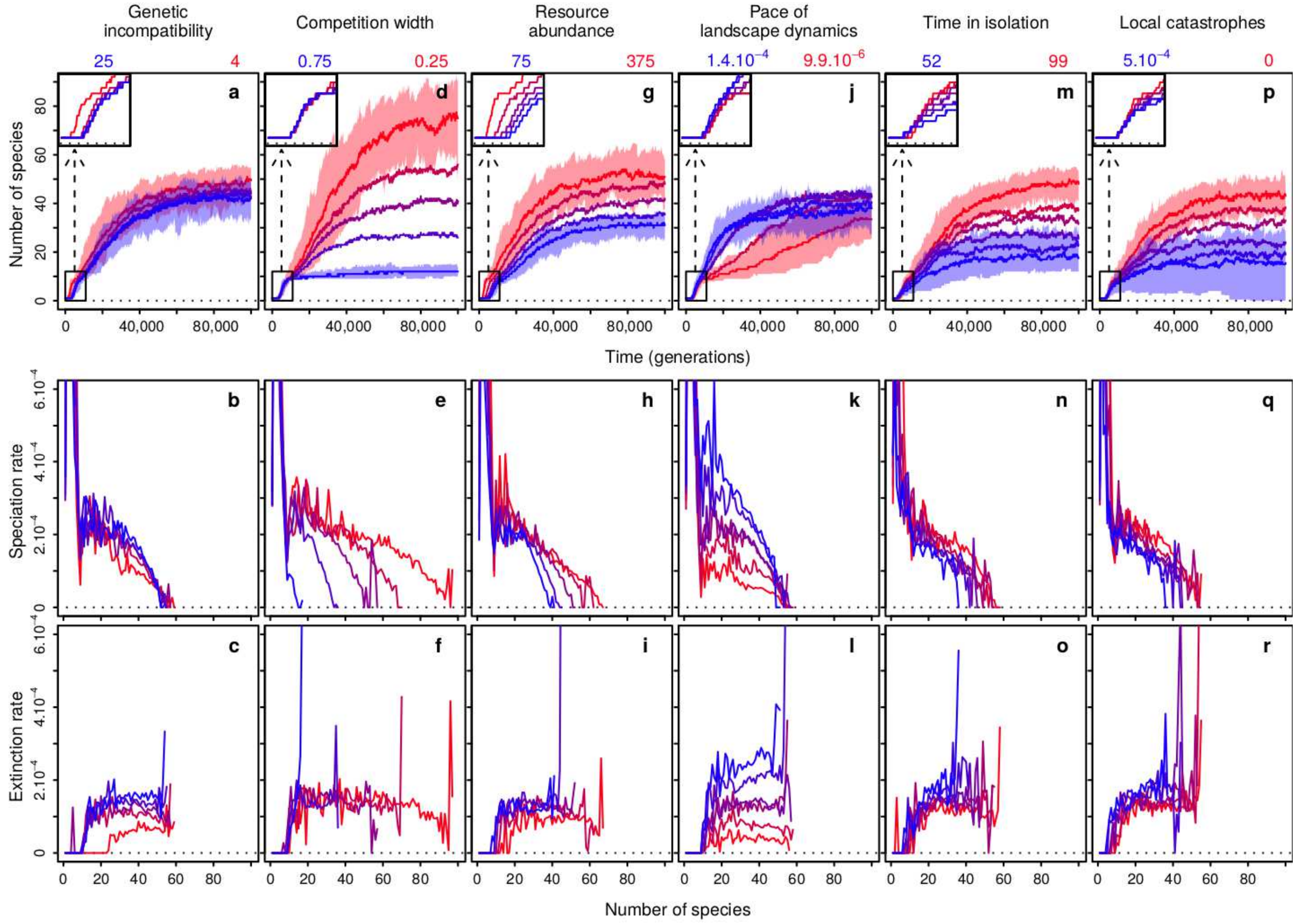


**Supplementary Figure 5: Effect of different biotic and abiotic factors on patterns of species diversification through time (first row), diversity-dependence of speciation rates (second row) and diversity-dependence of extinction rates (last row).** Parameter values varied for each factor (from left to right): *GIT* (number of genetic incompatibility loci causing reproductive isolation; **a-c**), $\sigma_C/\sigma_K$ (scaled competition width; **d-f**), *K** (carrying capacity at the environmental optimum; **g-i**), $1/(1/f + 1/c)$ (with *f* the rate of geographic barrier arising and *c* the rate of barrier removal; **j-l**), $f/(c + f)$ (**m-o**), and *cr* (rate of local catastrophic extinction; **p-r**). Results from different parameter values are shown on a blue to red gradient (with extreme values given above the plots), with blue (red) corresponding to lowest (highest) species diversity at stationary state. Median values (lines) and 95% confidence intervals (shaded areas) are based on 50 simulation replicates for each parameter set. Other parameter values as in Supplementary Table 1.

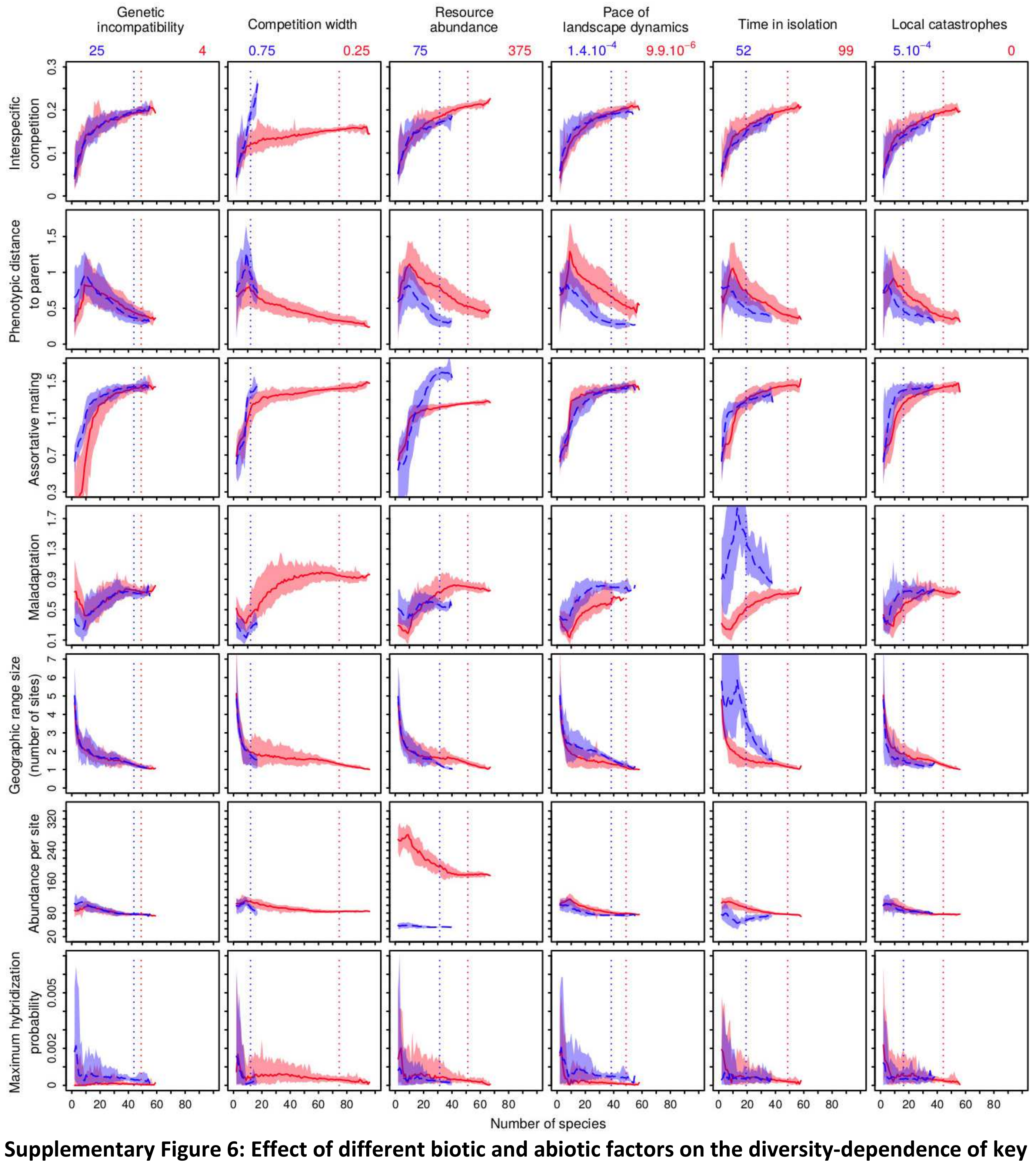


**Supplementary Figure 6: Effect of different biotic and abiotic factors on the diversity-dependence of key variables of macroevolutionary dynamics.** See Methods, section "2. Numerical simulations" for details about the computation of each variable. Parameter values varied for each factor (from left to right): *GIT* = 4 (red) *vs. GIT* = 25 (blue) (number of genetic incompatibility loci causing reproductive isolation; *first column*), $\sigma_C/\sigma_K$ = 0.25 (red) vs. $\sigma_C/\sigma_K$ = 0.75 (blue) (scaled competition width; *second column*), *K** = 375 (red) *vs. K** = 75 (blue) (carrying capacity at the environmental optimum; *third column*), $1/(1/f + 1/c) = 9.9.10^{-6}$ (red) *vs.* $1/(1/f + 1/c) = 1.4.10^{-4}$ (blue) (with *f* the rate of geographic barrier arising and *c* the rate of barrier removal; *fourth column*), $f/(c + f)$ = 99% (red) *vs.* $f/(c + f)$ = 52% (blue) (*fifth column*), and *cr* = 0 (red) *vs. cr* = $5.10^{-4}$ (blue) (rate of local catastrophic extinction; *sixth column*). Median values (lines) and 95% confidence intervals (shaded areas) are based on 50 simulation replicates for each parameter set. Other parameter values as in Supplementary Table 1.

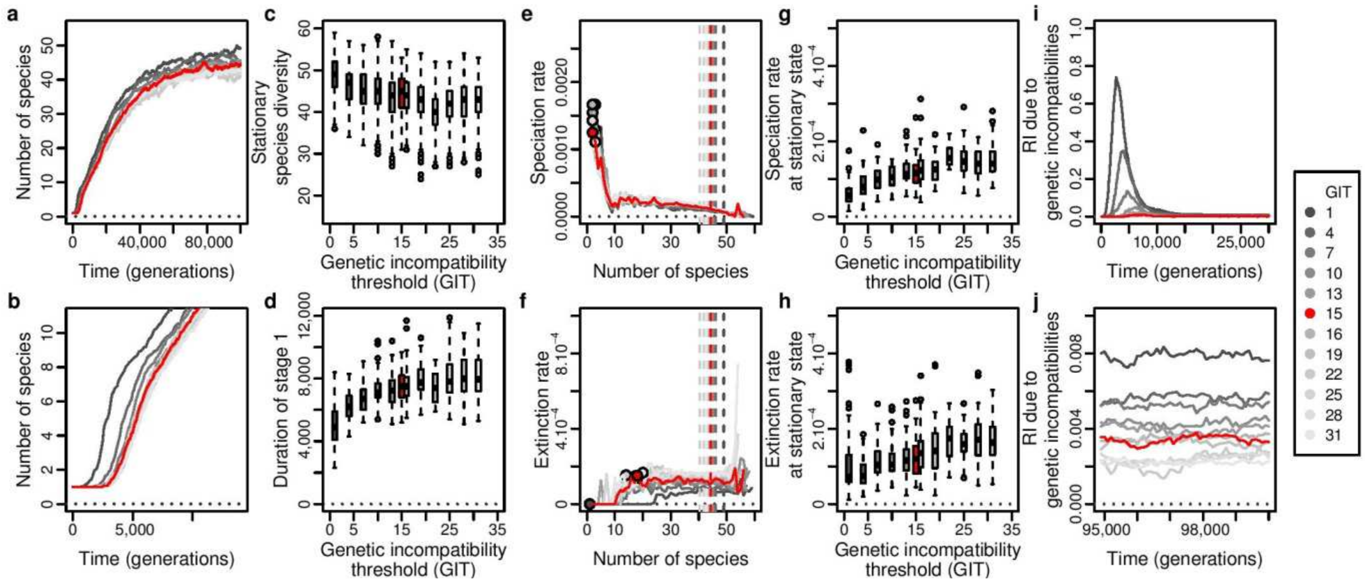


**Supplementary Figure 7: Influence of the number of genetic incompatibility loci causing reproductive isolation, *GIT*, on patterns of species diversification (a-d), diversity-dependence of macroevolutionary rates (e-f), macroevolutionary rates at stationary state (g-h), and mechanisms of reproductive isolation (RI) between populations (fraction due to genetic incompatibility as opposed to assortative mating; i-j).** Results in dark grey correspond to low *GIT*, results in light grey to high *GIT*, and red data correspond to *GIT* = 15 (that we adopted as default parameter value, Supplementary Table 1). Results are based on 50 simulation replicates, ran for 100,000 generations. Stationary state data were collected during the last 5,000 generations. Lines show mean values, and boxplots represent the first, second and third quartiles, with whiskers giving the maximum and minimum values. Dots on panels **e-f** highlight the maximum value of the trajectories restricted to species diversity below 20. Other parameter values as in Supplementary Table 1.

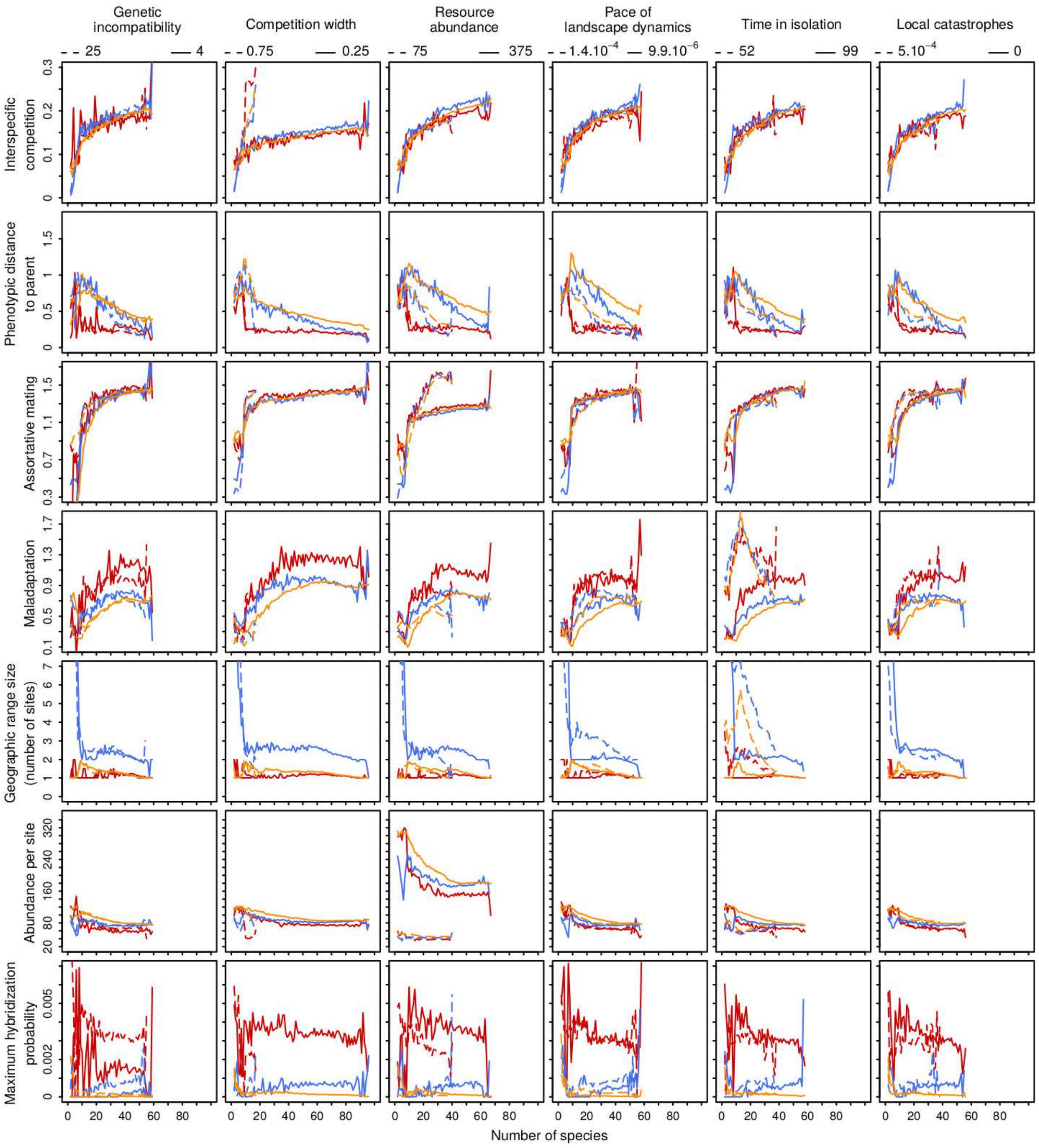


**Supplementary Figure 8: Effect of different biotic and abiotic factors on the diversity-dependence of key variables of macroevolutionary dynamics, for species that are about to speciate (within the next 200 generations; blue), species that are about to go extinct (within the next 200 generations; red), or 'static' species (that undergo neither speciation nor extinction within the next 2,000 generations; orange).** See Methods, section "2. Numerical simulations" for details about the computation of each variable. Parameter values varied for each factor (from left to right): *GIT* = 4 (plain lines) *vs. GIT* = 25 (dashed lines) (number of genetic incompatibility loci inducing reproductive isolation; *first column*), $\sigma_C/\sigma_K$ = 0.25 (plain lines) vs. $\sigma_C/\sigma_K$ = 0.75 (dashed lines) (scaled competition width; *second column*), *K** = 375 (plain lines) *vs. K** = 75 (dashed lines) (carrying capacity at the environmental optimum; *third column*), $1/(1/f + 1/c) = 9.9.10^{-6}$ (plain lines) *vs.* $1/(1/f + 1/c) = 1.4.10^{-4}$ (dashed lines) (with *f* the rate of geographic barrier appearance and *c* their rate of disappearance; *fourth column*), $f/(c + f)$ = 99% (plain lines) *vs.* $f/(c + f)$ = 52% (dashed lines) (*fifth column*), and *cr* = 0 (plain lines) *vs. cr* = $5.10^{-4}$ (dashed lines) (rate of local catastrophic extinction; *sixth column*). Lines show median values based on 50 simulation replicates for each parameter set. Other parameter values as in Supplementary Table 1.

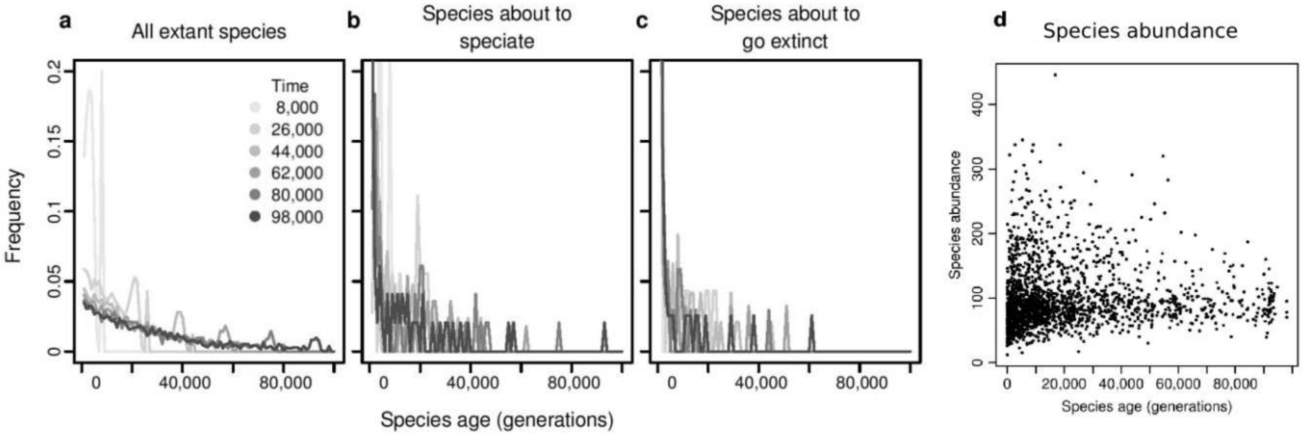


**Supplementary Figure 9: Distribution of species lifespan (a-c) and influence of species age on species abundance at stationary state (d).** Results are shown at different times during diversification (from light to dark grey, in number of generations), for (a) all extant species; (b) species that are about to speciate (within the next 200 generations; (c) species that are about to go extinct (within the next 200 generations (**c**). In panels **a-c** data are averaged over 50 simulation replicates. In panel **d**, data are shown at $t$ = 98,000, from 50 simulation replicates. The many species which are rare in a community include three groups: young ephemeral species which will soon go extinct, young species on a rapid growth trajectory, and old, chronically rare species. In all panels: parameter values as in Supplementary Table 1.

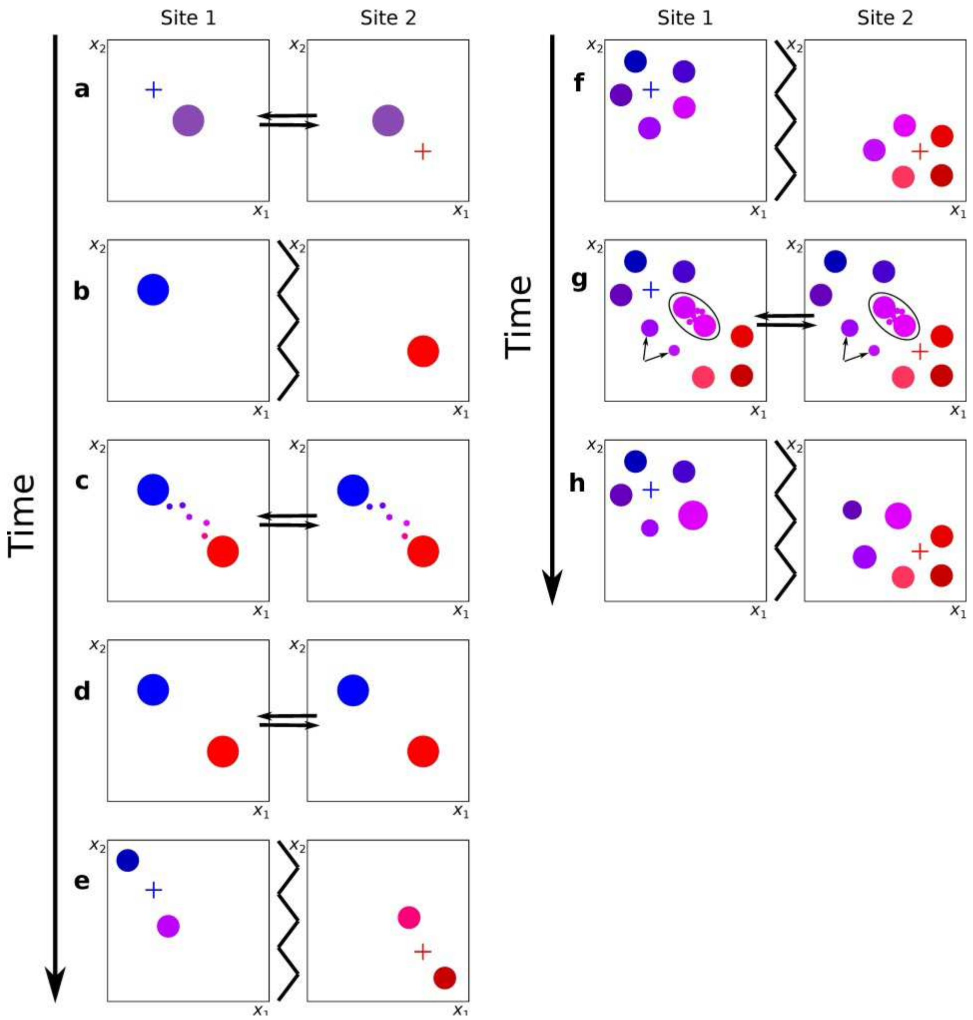


**Supplementary Figure 10: Competition, adaptation, and speciation in a dynamic landscape.** The birth of a new species requires that the mother species is distributed over two sites or more (**a**; circles show the range of phenotypes of living individuals). When two of these sites enter a bout of geographic isolation, the two populations may diverge through adaptation toward each local environmental optimum (**a**-**b**; local optimum is indicated by a '+' sign). Phenotypic divergence causes ecological speciation, potentially completed at secondary contact through reinforcement (evolution of stronger assortative mating, **c**-**d**). When sites are isolated again, the two incipient species may coexist in each site; then the two geographic pairs may differentiate themselves under the combined action, within each site, of intraspecific competition, which favors character convergence toward the local optimum, and interspecific competition, which limits similarity (**e**). Sites connection exposes poorly differentiated, single-site populations to competitive exclusion (**f**-**g**, arrows) and hybridization (**f**-**g**, oval), while opening opportunities for colonization and establishment of new populations that could become isolated and differentiated into new species in the future. Sites disconnection creates opportunities for the divergence of new species, while exposing poorly differentiated populations to the risk of local extinction by maladaptation (**g**-**h**).

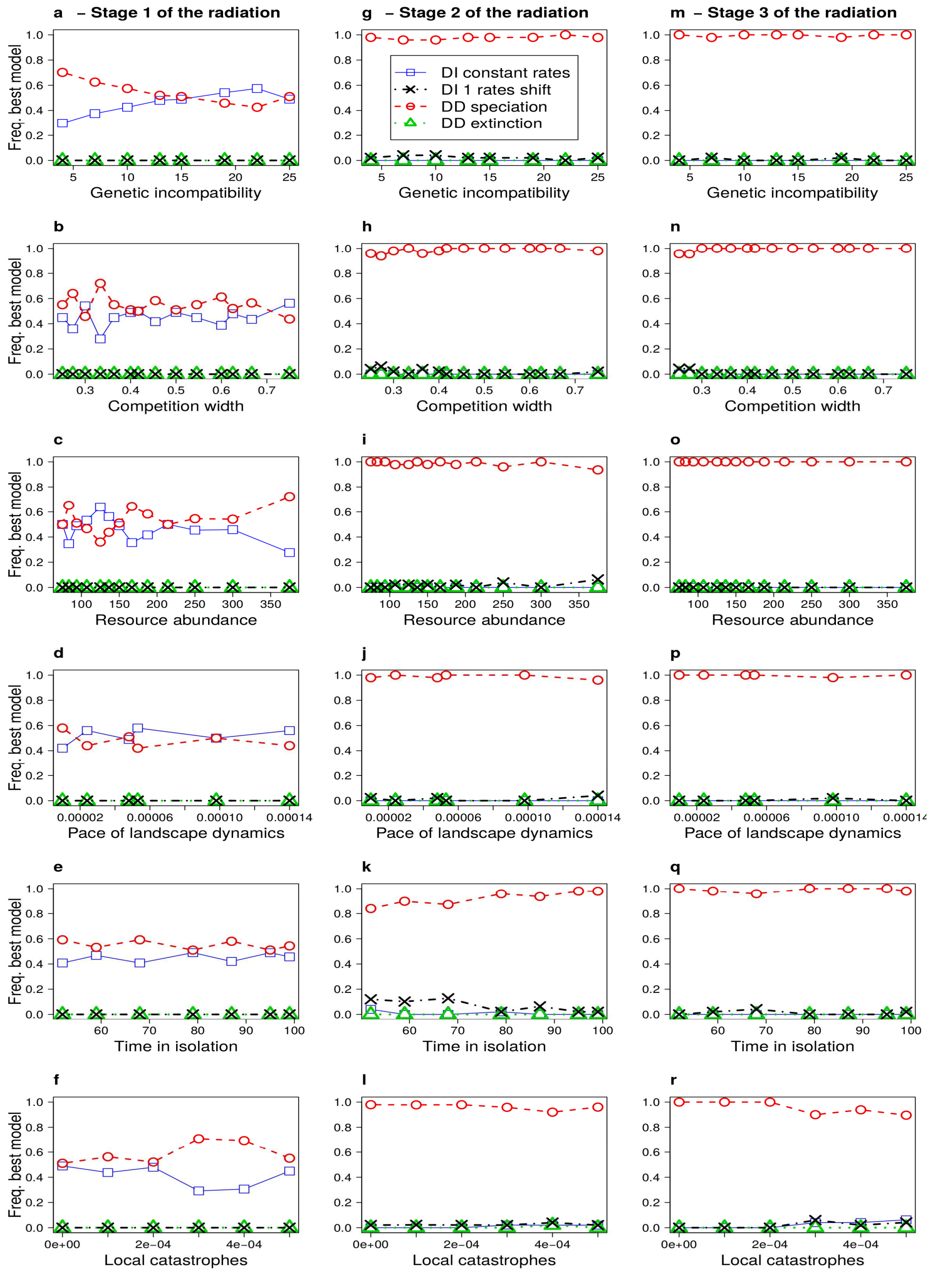


**Supplementary Figure 11: Frequency at which diversity-independent and diversity-dependent models of diversification rates are selected as the best model explaining macroevolutionary rates at the end of stage 1 (a-f), of stage 2 (g-l) and of stage 3 (m-r) of the radiation.** Four models are compared: constant speciation and extinction rates (“DI constant rates”, blue squares), constant speciation and extinction rates with 1 rates shift during diversification (“DI 1 rate shift”, black crosses), diversity-dependent speciation rate and constant extinction rate (“DD speciation”, red circles), diversity-dependent extinction rate and constant speciation rate (“DD extinction”, green triangles). The maximum likelihood of the models “DI constant rates”, “DD speciation” and “DD extinction” was computed using the R-package DDD[2] and the maximum likelihood of the model “DI 1 rates shift” was computed using the R-package TreePar[3]. The best model was then selected for each simulation replicate according to the AICc (stage 1; no more than 9 branches at this stage) or AIC (stages 2 and 3) criterion. For a few simulation replicates (maximum 6 replicates over 50 for each set of parameters value), the selected model depended on the initial conditions used to estimate the evolutionary rates. These replicates were discarded for computing the frequency of the best model. Each row show the effect of a biotic or abiotic factor (from top to bottom): number of genetic incompatibility loci causing reproductive isolation (*GIT*; first row), scaled competition width ($\sigma_C/\sigma_K$; second row), resource abundance (*K**; third row), pace of landscape dynamics ($1/(1/f + 1/c)$, with *f* the rate of geographic barrier arising and *c* the rate of barrier removal; fourth row), time in isolation ($f/(c + f)$; fifth row), and rate of local catastrophic extinction (*cr*; sixth row). Other parameter values as in Supplementary Table 1.

**Supplementary Table 1: Parameter notations and default values.**

| Parameter | Definition | Default value |
|---|---|---|
| $r$ | Per-capita birth rate | 1 |
| $L_k$ | Number of loci coding each trait $k$ ($k = x_1$, $x_2$ or $a$) | 16 |
| $\mu_k$ | Mutation probability at each locus coding for trait $k$ ($x_1$, $x_2$ or $a$) | $10^{-3}$ |
| $\boldsymbol{s_x^2}$ | Expected phenotypic variance of phenotypic traits $x_1$ and $x_2$ | $1.6.\ 10^{-3}$ |
| $\boldsymbol{s_a^2}$ | Expected phenotypic variance of choosiness trait $a$ | 0.01 |
| $c_{\mathrm{am}}$ | Coefficient in the assortative mating function | 10 |
| *AMT* | Assortative mating threshold (cross-breeding probability determining pre-zygotic reproductive isolation) | 0.01 |
| $\mu_{\mathrm{n}}$ | Mutation probability at loci determining genetic incompatibility | $10^{-3}$ |
| *GIT* | Genetic incompatibility threshold (number of genetic incompatibility loci involved in post-zygotic reproductive isolation) | 15 |
| $K^*$ | Carrying capacity at the environmental optimum in each site | 150 (individuals) |
| $\sigma_{\mathrm{K}}$ | Standard deviation of the carrying capacity function in each site | 1.0 |
| $\sigma_{\mathrm{C}}$ | Standard deviation of the competition kernel | 0.4 |
| $n^2$ | Number of sites in the landscape | 9 |
| $\delta_{\mathrm{x}}$ | Difference in optimal phenotypic trait values between adjacent sites | 1.0 |
| $f$ | Rate of geographic barrier arising | $10^{-3}$ |
| $c$ | Rate of geographic barrier removal | $5.10^{-5}$ |
| *cr* | Rate of local catastrophic extinction | 0 |

## Supplementary References


1. Gascuel, F., Ferrière, R., Aguilée, R. & Lambert, A. How ecology and landscape dynamics shape phylogenetic trees. *Syst Biol* **64,** 590–607 (2015).
2. Etienne, R. S. *et al.* Diversity-dependence brings molecular phylogenies closer to agreement with the fossil record. *Proc R Soc Lond B* **279,** 1300–1309 (2012).
3. Stadler, T. Mammalian phylogeny reveals recent diversification rate shifts. *Proc Natl Acad Sci USA* **108,** 6187–6192 (2011).
