# Supplementary material for: Clade diversification dynamics and the biotic and abiotic controls of speciation and extinction rates
Source: Nat Commun. 2018 Aug 1;9:3013. doi: 10.1038/s41467-018-05419-7 (PMC6070539; doi:10.1038/s41467-018-05419-7)
Supplement: Supplementary file 3 — Description of Additional Supplementary Files [file 41467_2018_5419_MOESM3_ESM.pdf]

Description of Additional Supplementary Files

File Name: Supplementary Data 1
Description: Compressed file containing the source code for running the simulations and the analyses.
